# Transduction Efficiency and Immunogenicity of Viral Vectors for Cochlear Gene Therapy: A Systematic Review of Preclinical Animal Studies

**DOI:** 10.3389/fncel.2021.728610

**Published:** 2021-08-30

**Authors:** Dorien Verdoodt, Noa Peeleman, Guy Van Camp, Vincent Van Rompaey, Peter Ponsaerts

**Affiliations:** ^1^Department of Translational Neurosciences, Faculty of Medicine and Health Sciences, University of Antwerp, Antwerp, Belgium; ^2^Laboratory of Experimental Hematology, Vaccine and Infectious Disease Institute (Vaxinfectio), University of Antwerp, Antwerp, Belgium; ^3^Department of Medical Genetics, Faculty of Medicine and Health Sciences, University of Antwerp, Antwerp, Belgium; ^4^Department of Otorhinolaryngology and Head & Neck Surgery, Antwerp University Hospital, Antwerp, Belgium

**Keywords:** transduction efficiency, inner ear, adeno-associated virus, gene therapy, immunogenicity

## Abstract

**Background:** Hearing impairment is the most frequent sensory deficit, affecting 466 million people worldwide and has been listed by the World Health Organization (WHO) as one of the priority diseases for research into therapeutic interventions to address public health needs. Inner ear gene therapy is a promising approach to restore sensorineural hearing loss, for which several gene therapy applications have been studied and reported in preclinical animal studies.

**Objective:** To perform a systematic review on preclinical studies reporting cochlear gene therapy, with a specific focus on transduction efficiency.

**Methods:** An initial PubMed search was performed on April 1st 2021 using the PRISMA methodology. Preclinical *in vivo* studies reporting primary data regarding transduction efficiency of gene therapy targeting the inner ear were included in this report.

**Results:** Thirty-six studies were included in this review. Transduction of various cell types in the inner ear can be achieved, according to the viral vector used. However, there is significant variability in the applied vector delivery systems, including promoter, viral vector titer, etc.

**Conclusion:** Although gene therapy presents a promising approach to treat sensorineural hearing loss in preclinical studies, the heterogeneity of methodologies impedes the identification of the most promising tools for future use in inner ear therapies.

## Introduction

Hearing loss and balance loss have a significant impact on quality of life and society in general. Hearing impairment is among the most frequent sensory deficits in human populations, affecting 440 million people worldwide and has been listed by the World Health Organization (WHO) as one of the priority diseases for research into therapeutic interventions to address public health needs (WHO, 2013; Davis and Hoffman, 2019). Currently, no disease-modifying therapies are available to slow down or prevent progressive sensorineural hearing loss from happening in humans (Yoshimura et al., 2019). Instead, treatment is currently focused of hearing rehabilitation, which means fitting hearing aids that amplify sounds in case of moderate-to-severe sensorineural hearing loss (Hoppe and Hesse, 2017; Suen et al., 2019). In case of severe-to-profound sensorineural hearing loss, cochlear implantation provides a solution by electrically stimulating spiral ganglion neurons (Bond et al., 2009, 2010; Landsberger et al., 2016; Vickers et al., 2016). However, emerging alternatives that could prevent hearing loss or restore hearing permanently are based on gene therapy and are considered to become part of successful future therapeutic interventions.

The first-in-human phase 1/2 clinical gene therapy trial (NCT02132130, conducted in the US) has been aiming to upregulate the atonal gene (ATOH1/MATH1) in supporting cells of the inner ear and to trigger their trans-differentiation into functional hair cells (Praetorius et al., 2009; Omichi et al., 2019; Ren et al., 2019). Recently reported rodent studies on gene replacement and gene editing therapy have generally been aiming to restore hearing in case of congenital sensorineural hearing loss by recovery of gene and protein expression, and subsequent restoration of sensory cell function (Iizuka et al., 2015; Emptoz et al., 2017; Pan et al., 2017; Akil et al., 2019b; Taiber et al., 2021). Gene editing strategies have also been explored in autosomal dominant disorders (which mainly involve single nucleotide substitutions) to disrupt dominant mutations selectively without affecting wild-type alleles (Gao et al., 2018; Gyorgy et al., 2019).

However, preclinical studies reporting outcome can be quite different in their study design when looking at the species and strains that were studied, the number of animals (including gender), the applied vector type as well as its titer and route of administration, the reporter and promoter genes used, histological assessment of transduction efficiency, etc. In contrast to the clinical field, systematic reviews are rare within preclinical animal research in general, and non-existent in the field of cochlear gene therapy more specifically. Nonetheless, preclinical animal research is the foundation for (future) clinical trials and their study design (Mignini and Khan, 2006; Peters et al., 2006; Leenaars et al., 2012). Therefore, a key aspect to translate preclinical research to human trials is safety of the intervention. In the context of inner ear gene therapy, the question arises to what extent this methodological intervention is able to transduce a sufficiently high number of target cells, displays immunogenicity and/or has itself an effect on hearing thresholds. In this context, it is of utmost importance that administration of the vector does not have any detrimental side effects, e.g., aggravation of hearing or balance impairment. Although several studies have reported that gene delivery using adeno-associated viral vectors (AAV) caused minimal changes in the threshold of auditory brain stem recordings (ABR), some studies have observed significant threshold shifts, often the result of the delivery method (Chien et al., 2015; Yoshimura et al., 2018). However, there are only few studies that perform in-depth immunogenicity and/or functional studies using the targeting vector itself.

The objective of this study is to provide a systematic literature overview to summarize minimal criteria to determine preclinical safety and immunogenicity of viral vector administration in animal models of hearing loss.

## Methodology and Results

This systematic review was based on the methodology of PRISMA (Preferred Reporting Items for Systematic Review and Meta-Analysis) (Hooijmans et al., 2014). The search was performed April 1st 2021 in PubMed using the search term (“cochlea”[MeSH Terms] OR “cochlea”[All Fields]) AND {“viral vector”[All Fields] OR AAV[All Fields] OR (“genetic vectors”[MeSH Terms] OR (“genetic”[All Fields] AND “vectors”[All Fields]) OR “genetic vectors”[All Fields] OR “vector”[All Fields] OR “disease vectors”[MeSH Terms] OR (“disease”[All Fields] AND “vectors”[All Fields]) OR “disease vectors”[All Fields])}. Retrieved studies dated from the late 1980s until the search date.

**Figure 1:**
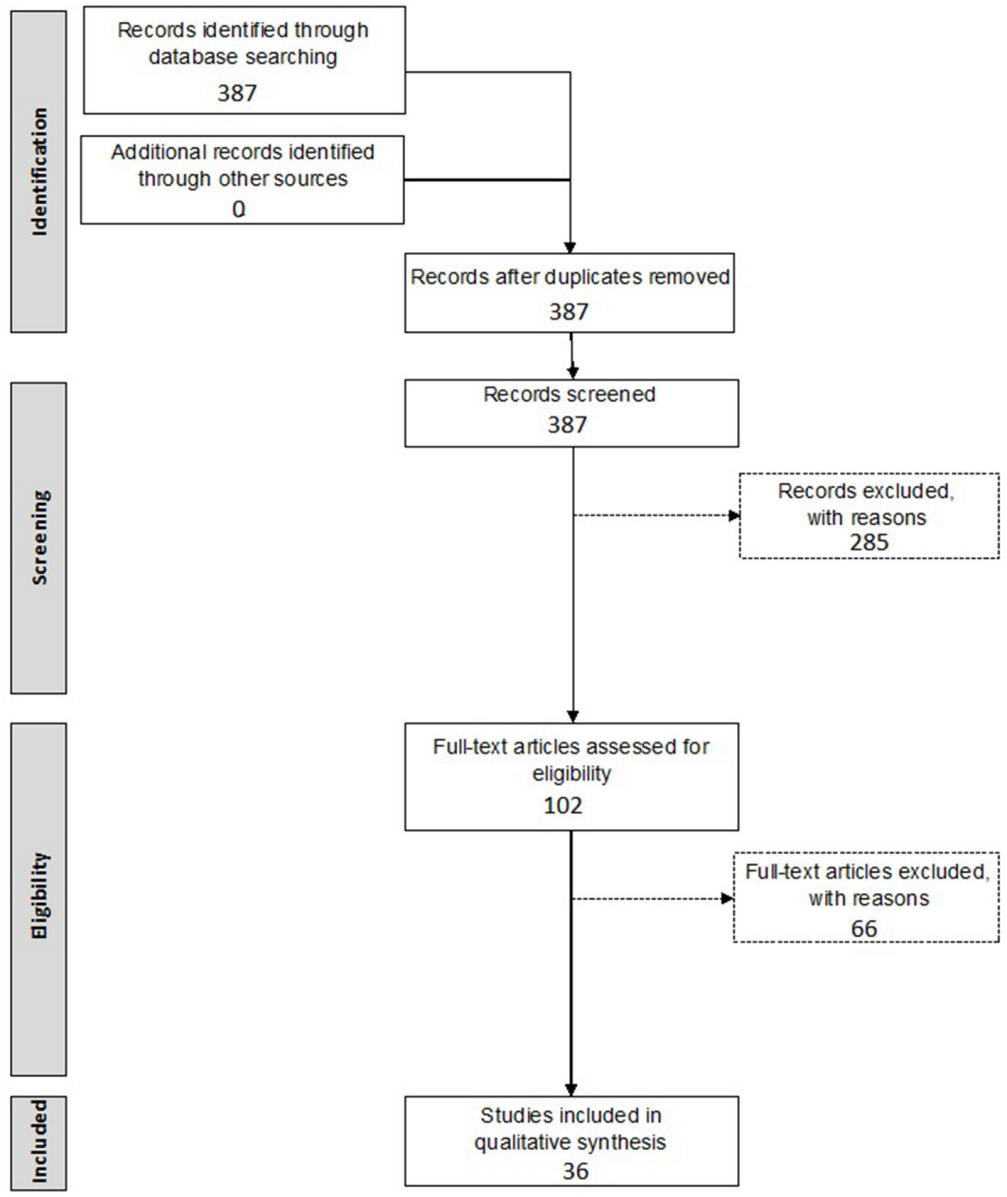
Flow chart of systematic review of gene therapy in the inner ear.

A first inclusion selection was based on the title and abstract of the retrieved records, while the second inclusion selection was based on the actual data provided within the full-text manuscript. All relevant data, as indicated below, were extracted independently by two investigators (NP and DV), after which discrepancies were discussed until consensus was reached. All steps of the screening procedure are presented in Figure 1.

Inclusion criteria included: (1) preclinical animal studies, (2) involving gene therapy, (3) introduced into the inner ear, (4) by means of a viral vector. Exclusion criteria included: (1) if written in a language other than English, (2) studies not reporting on primary data such as reviews, perspective papers, letters to the editor, etc., (3) incorrect study design/intervention, (4) no reporting on *in vivo* transduction efficiency or (5) no *in vivo* experiments performed. The initial search resulted in 387 papers. After screening by title and abstract, 285 papers were excluded. The remaining 102 studies were screened in full-text. Finally, 36 articles were included in this systematic review.

The following data were extracted from each record: title, authors, journal of publication, species and strain, number of animals, vector type, reporter, promoter, vector titer, volume and route of administration, transduction efficiency, effect on hearing level and a potential inflammatory response. The extracted data are summarized in Tables 1–3.

**Table 1:** Summary of extracted data from gene therapy studies performed in mice.

**Strain**	**Age**	**Vector**	**Reporter**	**Promoter**	**n**	**Route**	**Injected dose**	Transduction efficiency^*^					**Hearing**	**Immune**	**Ref**
								**IHC**	**OHC**	**SGN**	**SC**	**Other**			
BALB/c	5 w	Ad5	GFP	CMV	4	TUMI	5 x 10^6^ ffu		x	x	x	SV, SLb, RM	+^**^	+	Sheffield et al. (2011)
		Adf.11D	GFP		9	TUMI	5,35 x 10^6^ ffu			x					
		BovineAAV	GFP	CMV	8	TUMI	2,5 x 10^7^ DRP	x	x	51.1%	PC, ISC, OSC				
C3Hfe	P15–16	AAV2/9	GFP	CMV	4	RWM+CF	3.30 x 10^10^ vg	99.42%					+	+	Yoshimura et al. (2019)
C3Hfe	P15–16	AAV2/Anc80L65	GFP	CMV	/	RWM+CF	1.4 x 10^9^ vg	89.07%					+	-	Yoshimura et al. (2018)
		AAV2/9	GFP	CMV	/	RWM	3.9 x 10^10^ vg	30.27%							
		AAV2/9	GFP	CMV	/	RWM+CF	3.9 x 10^10^ vg	94.27%	Limited						
		AAV2/9	GFP	CMV	/	RWM+CF	1.4 x 10^9^ vg	17.37%							
C3Hfe	Neonatal	rAAV2/9	GFP	CMV	6	STVI	1,64 x 10^12^ vg	96%		79.33%			+	-	Shibata et al. (2017)
		rAAV2/9	GFP	CMV	8	STVI	3,28 x 10^11^ vg	30%							
C57BL/6J	P1	AAV2/1-WPRE	GFP	CMV	5	RWM	1 x 10^10^ gc	Moderate to high	<5%				+	-	Landegger et al. (2017)
		AAV2/2-WPRE	GFP	CMV	4	RWM	1 x 10^10^ gc	Low	<5%						
		AAV2/6-WPRE	GFP	CMV	1	RWM	1 x 10^10^ gc	Low	<5%						
		AAV2/8-WPRE	GFP	CMV	2	RWM	1 x 10^10^ gc	Low	<5%						
		AAV2/Anc80L65-WPRE	GFP	CMV	3	RWM	1 x 10^10^ gc	100%	90%						
C57BL/6J	P0–P30	AAV-ie-WPRE	mNeon Green	CAG	/	RWM	3.6 x 10^9^ gc	Almost all	Most		76.17%		+	-	Tan et al. (2019)
		AAV1-WPRE	mNeon Green	CAG	/	RWM	3.6 x 10^9^ gc				<20%				
		AAV6-WPRE	mNeon Green	CAG	/	RWM	3.6 x 10^9^ gc				<20%				
		AAV8-WPRE	mNeon Green	CAG	/	RWM	3.6 x 10^9^ gc				<20%				
		AAV9-WPRE	mNeon Green	CAG	/	RWM	3.6 x 10^9^ gc				<20%				
		AAV-PHP.eB-WPRE	mNeon Green	CAG	/	RWM	3.6 x 10^9^ gc				<20%				
		Anc80L65-WPRE	mNeon Green	CAG	/	RWM	3.6 x 10^9^ gc				<55%				
		AAV-DJ-WPRE	mNeon Green	CAG	/	RWM	3.6 x 10^9^ gc				<55%				
	4 w	AAV-ie-WPRE	mNeon Green	CAG	/	RWM	1 x 10^10^ gc				57.33%				
C57BL/6J	P0–14	AAV2/1-WPRE	Tmc1	CMV	/	RWM	8.1 x 10^11^ gc						+	-	Nist-Lund et al. (2019)
		AAV2/Anc80-WPRE	GFP	CMV	/	RWM	1.4 x 10^10^ gc								
		AAV2/Anc80-WPRE	Tmc1ex1	CMV	4	RWM	1.4 x 10^11^ gc	91%	50%						
		AAV2/Anc80-WPRE	Tmc2	CMV	/	RWM	1.6 x 10^11^ gc								
C57BL/6J	P7	rAAV8-mut733	GFP	CBA	8	RWM+coll	4.5 x 10^9^ gc	48.35%	15%	Low			+	-	xia et al. (2012)
		rAAV8-mut733	GFP	CBA	6	RWM	4.5 x 10^9^ gc	54.50%	18.50%	Low	x	SLb, MT			
C57BL/6J	P0–1	AAV2/1	Gjb2	CB7	/	RWM	5.25 x 10^9^ gc						+	-	Yu et al. (2014)
		AAV2/1	GFP	CB7	8	RWM	4.2 x 10^8^ gc	29.67%		HeC: 10% CC: 28.67% OSC: 98.33%	MC: 32.67% SSC: 44.67%				
C57BL/6J	10 w	AAV1	GFP	CMV	>3	PSC	2.7 x 10^10^ gc	7.57%	0		x		+^**^	-	Tao et al. (2018)
		AAV2	GFP	CBA	>3	PSC	1.5 x 10^10^ gc	85.54%	8.7%		x				
		AAV6.2	GFP	CBA	>3	PSC	1.2 x 10^10^ gc	5.56%	0		x				
		AAV8	GFP	CBA	>3	PSC	1.35 x 10^10^ gc	72.3%	5.5%		x				
		AAV9	GFP	CBA	>3	PSC	1.5x10^10^ gc	62.71%	0		x				
		AAVrh.39	GFP	CBA	>3	PSC	1.5 x 10^10^ gc	53.24%	0						
		AAVrh.43	GFP	CBA	>3	PSC	9 x 10^9^ gc	94.8%	0		HeC				
		AAV2/Anc80L65	GFP	CMV	>3	PSC	8.4 x 109 gc	92.2%	39.09%						
		Ad5	GFP	CMV	4	PSC	6 x 10^9^ pfu	Some loss	Nearly complete loss						
C57BL/6J	Neonatal	AAV9-PHP.B -WPRE	GFP	CBA	15	RWM	5 x 10^10^ vg	70%	45%				-	-	Gyorgy et al. (2018)
	4 w	AAV9-PHP.B -WPRE	GFP	CBA	/	PSC	2 x 10^10^ vg	Almost all	None						
CD1	Neonatal	AAV9-PHP.B-WPRE	GFP	CBA	5	RWM	5 x 10^10^ vg	70%	55%						
C57B/6J	P0–30	AAV2/1	GFP	CMV	5	RWM	3.94 x 10^9^ gc	10.2%	32%		HeC: 12.2% CC: 78.9% OSC: 57.8%	MC: 59.2% IC: 61.3%	+	+	Wang et al. (2013)
		AAV2/1	GFP	CB7	4	RWM	3.34 x 10^9^ gc			48.3%	HeC: 92.1% CC and OSC	ID: 62.7% MC and IC			
		AAV2/7	GFP	CMV	5	RWM	2.14 x 10^9^ gc	82.1%		57.2%		MC: 62.4% ID: 64.5%			
		LV	GFP	CMV	4	RWM	4 x 10^5^ gc				HeC	MC and IC			
		LV	GFP	Ubiquitin	4	RWM	4 x 10^5^ gc				HeC	MC and IC			
FVB	1–3 m	AAV2-OtofNT	GFP	CBA	8	RWM	1.26 x 10^10^ vg	77%					+	-	Akil et al. (2019b)
		AAV2-OtofCT	GFP	CBA	8	RWM	9 × 10^9^ vg								
CBA/Caj	2–12 m	AAV1	GFP	CMV	4	SM	2.16 x 10^9^ gc	63.6%		42.9%		RC	+^**^	-	Kilpatrick et al. (2011)
		AAV2	GFP	CMV	4	SM	1.03 x 10^9^ gc	90.6%		41.9%		RC, SL			
		AAV5	GFP	CMV	4	SM	1.71 x 10^9^ gc	50%		22.2%		RC			
		AAV6	GFP	CMV	4	SM	2.42 x 10^9^ gc	82%		37.5%		RC			
		AAV8	GFP	CMV	4	SM	3.22 x 10^9^ gc	94.3%		60%		RC, SL			
CBA/CaJ	6 w	AAV2/Anc80L65	GFP	CMV	5	PSC	9.6 x 10^8^ gc	100%	x	10%		RM, ID, SL, RC	+	-	Suzuki et al. (2017)
CBA/J	P0–5	AAV2.7m8	GFP	CAG	8	PSC	9.75 x 10^9^ gc	84.13%	83.03%		IPC: 86.1% IphC: 61.4%		+^**^	+^**^	Isgrig et al. (2019)
		AAV8BP2	GFP	CAG	13	PSC	1.10 x 10^10^ gc	55.7%	44.1%						
		AAV2	GFP	CAG	3	PSC	5.69 x 10^9^ gc	43.6%	54.5%						
		AAV8	GFP	CAG	4	PSC	1.166 x 10^10^ gc	86.0%	51.7%						
		Anc80L65	GFP	CAG	7	PSC	1.89 x 10^10^ gc	94.0%	67.0%						
CD1	Neonatal	AAV2/2-WPRE	GFP	CBA	3	SM	2 x 10^9^ vg	Few			Few		+	-	Gu et al. (2019)
		AAV2/9-WPRE	GFP	CBA	3	SM	2 x 10^9^ vg	56.87%	14.9%		17.87%				
		AAV2/Anc80L65-WPRE	GFP	CMV-β-globin	3	SM	4.16^8^ vg	100%	90.23%		24.33%				
CD1	P0–1	AAV1	GFP	CBA	23^a^	RWM	5 x 10^9^ gc	65%	14%				-	-	Gyorgy et al. (2017)
		AAV1	GFP	CBA	38^b^	CS	5 x 10^9^ gc	36%	17%	x	CC, HeC, ISC				
		exo-AAV1	GFP	CBA	23^a^	RWM	5 x 10^9^ gc	88%	25%						
		exo-AAV1	GFP	CBA	38^b^	CS	5 x 10^9^ gc	63%	28%	x	CC, HeC, ISC				
C57BL6 (LHFPL5 KO)	P1–2	exo-AAV1	GFP	CBA	4	RWM	2 x 10^11^ gc	72%	30%						
CD1B6F1	P6–7	AAV2/6-eGFP	GFP	hbA	7	RWM	1.44 × 10^10^ vg	77%	x	x	x		+	-	Al-Moyed et al. (2019)
		Otoferlin dual AAV2/6-TS-WPRE	GFP	hbA	10	RWM	1.2 × 10^10^ vg	30%							
		Otoferlin dual AAV2/6-Hyb-WPRE	GFP	hbA	9	RWM	1.38 × 10^10^ vg	19%							
FVB	P1–3	AAV5	GFP	CBA	/	RWM	1.4 x 10^11^ gc	80%	<1%	60%	x		+^**^	-	Akil et al. (2019a)
		AAV5	hGDNF	CBA	/	RWM	1.8 x 10^11^ gc								
whirler	P1–5	AAV2/8	GFP	CMV	8	RWM	5 x 10^9^ gc	14.43%					+^**^	-	Chien et al. (2016)
C57/FVB	P1–3 P10–12	AAV1-VGLUT3 AAV1-VGLUT3 AAV1-VGLUT3	GFP GFP GFP	CBA CBA CBA	/ / /	RWM RWM RWM	1.38 x 10^10^ gc 2.3 x 10^10^ gc 1.38 x 10^10^ gc	100% 100% 40%					+	-	Akil et al. (2012)
C57BL/6	P0–2	AAV2/1 AAV2/1 AAV2/1–Tmc1 AAV2/1-Tmc2	GFP GFP GFP GFP	CBA CMV CBA CBA		RWM RWM RWM RWM	6 x 10^9^gc 4.4 x 10^9^ gc 2.4 x 10^10^ gc 1.8 x 10^10^ gc	59% 9% 64.5%	4%				+	-	Askew et al. (2015)
C3H/FeJ	4 w	AAV1 AAV2 AAV8 AAV9 Anc80 AAV2-mCherry AAV9-mCherry and AAV9	GFP GFP GFp GFP GFp GFP GFP	CMV CMV CMV CMV CMV CMV CMV	6 4 4 6 5 4 3	RWM + CF RWM + CF RWM + CF RWM + CF RWM + CF RWM + CF RWM + CF	3.75 x 10^9^ gc 3.68 x 10^9^ gc 4.94 x 10^9^ gc 1.20 x 10^9^ gc 5.5 x 10^9^ gc 1.07 x 10^9^ gc 1.24 x 10^9^ gc	8.2% 96.7% 85.1% 85.1% 99.5% 96.9% 28%	<5% 83.9% <5% <5% 43% 65.6% <5%				+	-	Omichi et al. (2020)

***Age**: P, days post-natal; w, weeks. **Vector**: AAV, adeno-associated virus; Ad, adenovirus; LV, lentivirus. **Reporter**: GFP, green fluorescent protein. **Promoter**: CMV, cytomegalovirus; CAG, hybrid promoter; CB7, hybrid promoter; CBA, chicken β-actin; hbA, human β-actin. **n-value**: / = unknown*,

a *23 animals were used in total for the RWM approach*,

b *38 animals were used in total for the cochleostomy. **Route**: TUMI, transuterine microinjection; RWM, round window membrane; CF, canal fenestration; STVI, superior temporal vein injection; coll, collagenase; PSC, posterior semicircular canal; SM, microinjection in scala media; CS, cochleostomy. **Dose/Volume**: pfu, plaque-forming unit; ffu, focal forming unit; DRP, Dnase resistant particles; vg, viral genomes; gc, genome copies; ml, milliliter; μl, microliter. **Transduction efficiency**: IHC, inner hair cell; OHC, outer hair cell; SGN, spiral ganglion neuron; SC, supporting cell in organ of Corti; IPC, inner pillar cell; OPC, outer pillar cell; PC, pillar cell; HeC, hensen's cell; ID, interdental cell; ISC, inner sulcus cell; OCS, outer sulcus cell; CC, claudius cell; MC, marginal cell; IPhC, inner phalangeal cell; IC, intermediate cell; SSC, spindle-shaped cell; SV, stria vascularis; SLb, spiral limbus; SL, spiral ligament; RM, reissner's membrane; MT, membrana tectoria; RC, rosenthal's canal*.

* *In case multiple slides were analyzed from basal to apical regions or multiple values were provided, mean values were calculated. **Hearing/Immune**: + indicates whether the study tested the effect on hearing/immune response compared to non-injected controls, - indicates this topic was not investigated or reported*.

** *Indicates a negative effect was reported*.

**Table 2:** Summary of extracted data from gene therapy studies performed in guinea pigs.

**Strain**	**Age**	**Vector**	**Reporter**	**Promoter**	**n**	**Route**	**Dose/Volume**	Transduction efficiency^*^					**Hearing**	**Immune**	**Ref**
								**IHC**	**OHC**	**SGN**	**SC**	**Other**			
Albino	2-3m	rAAV8-mut733	Myc tag	CBA	3	CS	6.92 x 10^11^	80%					+	-	Chen et al. (2018)
Albino	Adult	Ad5	GFP		8	RWM	5 x 10^7^			x	x		+	-	Yang et al. (2012)
Albino	Adult	rAAV2/2	GFP	CMV	6	RWM+coll	1.5 x 10^9^ vg	33.27%	2.33%	84.52%			+	+	Wang et al. (2012)
		rAAV2/2	GFP	CMV	6	CS	1.5 x 10^9^ vg	63.3%	7.3%	86.01%					
		rAAV-mut733	GFP	CMV	4	RWM+coll	1.13 x 10^11^ vg	78.80%	27.61%						
		rAAV-mut733	GFP	CMV	4	CS	1.13 x 10^11^ vg	84.97%	43.43%						
Albino	Adult	AAV2/2	GFP	CMV	5	CS	1 x 10^10^ gc	97.2%	47.9%		HeC		+^**^	-	Konishi et al. (2008)
	Adult	AAV2/1	GFP	CAG	5	RWM	/	66.42%					-	+	Leake et al. (2019)
PDH	Adult	Ad5	GFP	CMV	5	CS	2.2 x 10^8^ OPU				IPC, OPC, DC, HeC, ISC	ID	+^**^	-	Atkinson et al. (2012)
		Ad5-NT3	GFP	CMV	4	CS	6 x 10^7^ OPU				IPC, OPC, DC, HeC, ISC	ID			
		Ad5-BDNF	GFP	CMV	/	CS	8.6 x 10^7^ OPU								
PDH	Adult	Ad5	GFP	CMV	5	CS	2.2 x 10^8^ OPU	x	x		IPC, OPC, HeC, DC	ID	-	-	Wise et al. (2011)
		Ad5-NT3	GFP	CMV	5	CS	6 x 10^7^ OPU	x	x		IPC, OPC, HeC, DC	ID			
		Ad5-BDNF	GFP	CMV	/	CS	8.6 x 10^7^ OPU								

***Age**: m, months. **Vector**: AAV, adeno-associated virus; Ad, adenovirus; rAAV, recombinant adeno-associated virus. **Reporter**: GFP, green fluorescent protein. Promoter: CMV, cytomegalovirus; CAG, hybrid promoter; CBA, chicken β-actin. **n-value**: / = unknown. **Dose/Volume**: vg, viral genomes; gc, genome copies; OPU, optical particle units; ml, milliliter. **Route**: RWM, round window membrane; coll, collagenase; CS, cochleostomy. **Transduction efficiency:** IHC, inner hair cell; OHC, outer hair cell; SGN, spiral ganglion neuron; SC, supporting cell in organ of Corti; IPC, inner pillar cell; OPC, outer pillar cell; HeC, hensen's cell; ID, interdental cell; DC, deiters cell; ISC, inner sulcus cell*.

* *In case multiple slides were analyzed from basal to apical regions or multiple values were provided, mean values were calculated. **Hearing/Immune**: + indicates whether the study tested the effect on hearing/immune response compared to non-injected controls, - indicates this topic was not investigated or reported*.

** *Indicates a negative effect was reported*.

**Table 3:** Summary of extracted data from gene therapy studies performed in other species.

**Species**	**Age**	**Vector**	**Reporter**	**Promoter**	**n**	**Route**	**Dose/Volume**	Transduction efficiency^*^					**Hearing**	**Immune**	**Ref**
								**IHC**	**OHC**	**SGN**	**SC**	**Other**			
Cats	4–5 w	AAV5	GFP	CBA	/	RWM	1.4 x 10^12^ gc	x		7.5%	x		-	+	Leake et al. (2019)
		AAV5-GDNF		CBA	5	RWM	1.8 x 10^12^ gc								
		AAV2	GFP	CAG	/	RWM	2 x 10^10^ gc	x	x	7.5%	IPC, OPC				
		AAV2-hBDNF		CAG	5	RMW	3 x 10^10^ gc								
Cynomolgus	Adult	AAV9-PHP.B	GFP	CBA	1	RWM	1 x 10^11^ vg	None	None	None	None	None	-	+^**^	Ivanchenko et al. (2020)
monkey		AAV9-PHP.B	GFP	CBA	1	RWM	2 x 10^11^ vg	50%	65%						
		AAV9-PHP.B	GFP	CBA	2	RWM	3,5 x 10^11^ vg	100%	100%	x	IPhC, OPhC, PC, HeC, CC	Border cells, SL, SLb and RM			
		AAV9-PHP.B	GFP	CBA	2	RWM	7 x 10^11^ vg	100%	100%	x	IPhC, OPhC, PC, HeC, CC	Border cells, SL, SLb and RM			
Bama miniature	P21–28	AAV1	GFP	CMV	3	PSC	3 x 10^10^ vg			x		SLb	+^**^	-	Ji et al. (2019)
pigs		AAV1	GFP	CMV	3	RWM	3 x 10^10^ vg	35%							
Miniature pigs	Adult	AAV1	GFP	CAG	5	RMW	/	42.25%			HeC, IPC and OPC	SLb, SL	-	+	Shi et al. (2017)
Wistar rats	4 w	Ad5	GFP		12	RWM	5 x 10^12^ IFU	x	x	High		BM, SV	-	-	Lei and Han (2010)
		AAV2	GFP		4	RWM	5 x 10^12^ IFU	x	x			SV			
		LV	GFP		4	RWM	2 x 10^8^ IFU					BM, SV			

*Age: w, weeks; P, post-natal days. Vector: AAV, adeno-associated virus; Ad, adenovirus; LV, lentivirus. Reporter: GFP, green fluorescent protein. Promoter: CMV, cytomegalovirus; CAG, hybrid promoter; CBA, chicken β-actin. **n-value**: / = unknown. **Route**: RWM, round window membrane; PSC, posterior semicircular canal. **Dose/Volume**: vg, viral genomes; gc, genome copies; IFU, infectious units; ml, milliliter. **Transduction efficiency**: IHC, inner hair cell; OHC, outer hair cell; SGN, spiral ganglion neuron; SC, supporting cell in organ of Corti; IPC, inner pillar cell; OPC, outer pillar cell; PC, pillar cell; HeC, hensen's cell; CC, claudius cell; IPhC, inner phalangeal cell; OPhC, outer phalangeal cells; BM, basement membranes; SV, stria vascularis; SLb, spiral limbus; SL, spiral ligament; RM, reissner's membrane*.

* *In case multiple slides were analyzed from basal to apical regions or multiple values were provided, mean values were calculated. **Hearing/immune**: + indicates whether the study tested the effect on hearing/immune response compared to non-injected controls, − indicates this topic was not investigated or reported*.

** *Indicates a negative effect was reported*.

**Figure 2:**
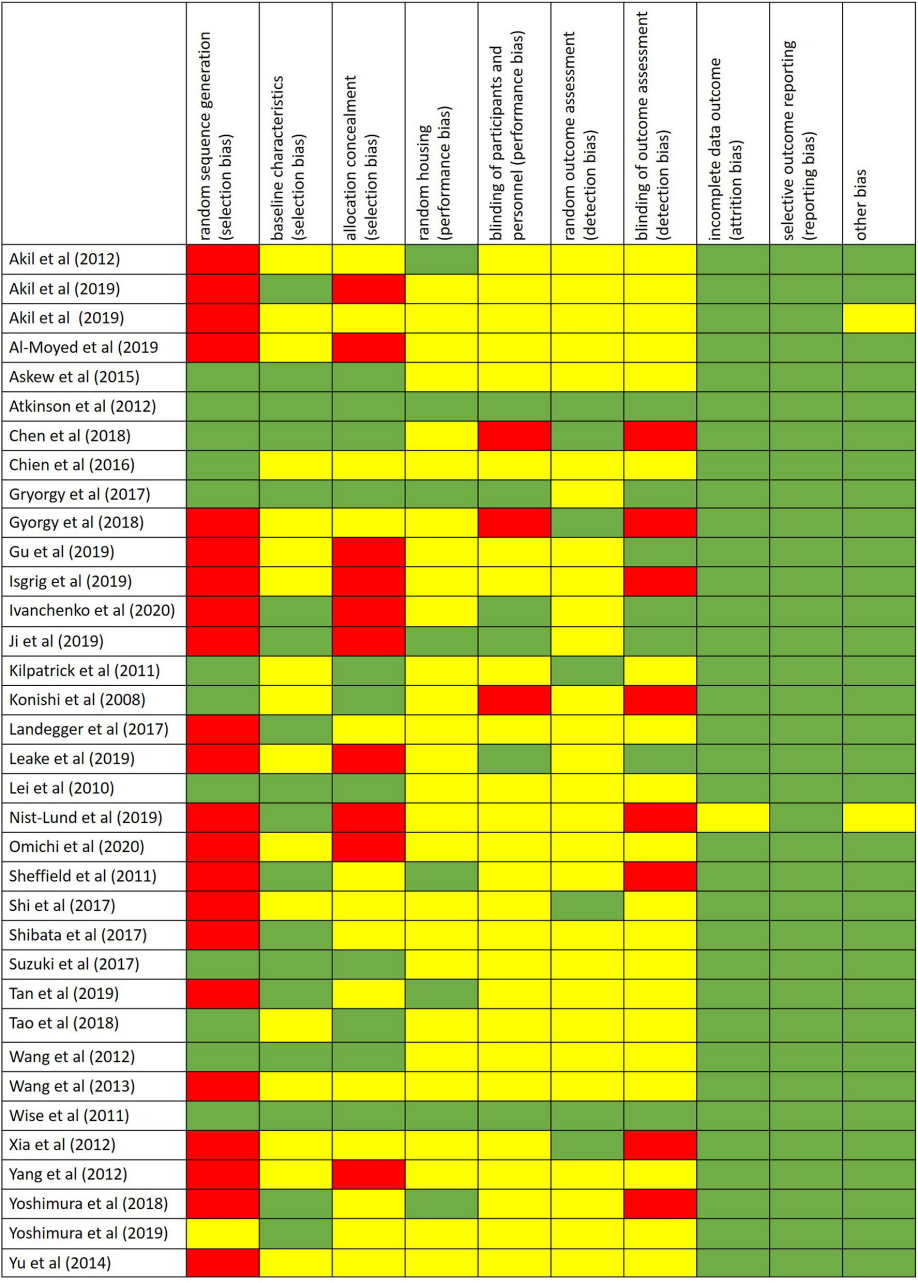
Risk of bias performed by using the SYRCLE's risk of bias tool for animal studies. Red = high risk of bias; Yellow = unknown risk of bias; Green = low risk of bias.

The risk of bias was assessed by using the SYRCLE's risk of bias tool for animal studies (Hooijmans et al., 2014) (Figure 2).

## Discussion

Several literature reviews have been published focusing on gene therapy in the inner ear. However, there are no systematic reviews on the use of gene therapy targeted at the inner ear in preclinical studies, with a specific focus on actual transduction efficiency. This systematic review identified a significant heterogeneity in preclinical studies when looking at all study characteristics, including the species and strains that were studied, the number of animals (inclusive gender) included, which vector type was used to introduce gene therapy, its titer and route of administration, the reporter and promoter genes used, as well as the actual transduction efficiency. This significant heterogeneity in study designs prevents researchers from performing a meta-analysis of previously performed experiments, and subsequently it is currently not possible to define the most optimal vector system for inner ear gene therapy. Nevertheless, several interesting concepts and/or critical reflections can be drawn from the provided tables and are briefly summarized and discussed below. These should allow to develop better experimental designs aiming to propose gene therapy methodological approaches that allow for reproducible *in vivo* transduction efficiency without adverse effects.

### Species

Several animal strains were used in the included studies, with mice being the most widely used species (*n* = 24), followed by guinea pigs (*n* = 7). Studies on rats (*n* = 1), miniature pigs (*n* = 2), cats (*n* = 1) and non-human primates (*n* = 1) were reported less commonly.

Currently, mice are the most widely used animal model in hearing research. Mice display a high level of similarity in genetics of human and mouse hearing (Ohlemiller, 2019). About 99% of mouse genes have a human ortholog and mutations in these genes often display similar deafness phenotypes compared to those associated with ortholog human mutations (Bowl and Dawson, 2015; Ohlemiller et al., 2016). However, an important difference to consider is the fact that the inner ear of mice needs to further develop postnatally and hearing onset usually occurs around P9-14 (Muller et al., 2019). This is important to keep in mind when choosing the age of mice in gene therapy research, as gene therapy in human will need to be performed in the developed hearing system. From the reported mice studies, experiments were performed, post-natal (P0-3, 52%), in infants (P3 up to 3 weeks, 24%), in juveniles (3–8 weeks, 12%), in adults (2–6 months, 8%) and in middle aged mice (6–12 months, 4%). Especially for gene replacement studies, early intervention is crucial in order to prevent developmental consequences (Ahmed et al., 2017) and this seems to be well-covered by preclinical mouse studies. However, in human clinical situation it may not be practical to perform gene therapy studies at the corresponding age as the injections have to be performed *in utero*. Therefore, future studies may need to additionally focus on similar therapeutic strategies in adult and middle-aged mice.

Another widely used animal model are guinea pigs. Guinea pigs display a great overlap with the audible frequency range in humans and their cochlea contains three and a half turns. This extra turn in guinea pigs can be useful in research using drugs that influence the cochlea (Reis et al., 2017; Naert et al., 2019). Other species that are frequently used for hearing research are rats, miniature pigs and non-human primates (NHPs). The cochlea of rats measures two and a half turn and they often suffer from otitis media as their tympanic membrane does not completely seal the external auditory canal and their Eustachian tube is horizontally orientated (Reis et al., 2017). Miniature pigs are also an important animal model when considering ontological research. They display numerous similarities with humans in both physiological functions and temporal bone structures. Furthermore, pig deafness models have more similarities with humans in both genetics and disease phenotypes compared to rodents (Ji et al., 2019). Finally, NHPs could also prove to be useful in hearing research as there are many similarities with humans, not only anatomical but also in terms of progressive hearing damage and many physiological processes and phenotypes associated with human disorders display high similarity to NHP models (Reis et al., 2017; Burton et al., 2019).

### Route of Administration

A challenge for gene therapy targeting the inner ear is the accessibility of the cochlea. Many studies have shown safe introduction of AAV into perilymphatic spaces without disruption of the barrier between endolymph and perilymph (Akil et al., 2015; Ji et al., 2019). The choice of delivery method used also depends on the target cell type (Ahmed et al., 2017). The round window membrane (RWM) approach is most frequently used as reported in 18 of the included studies. The RWM is the only soft-tissue access to the inner ear and is already widely used in cochlear implantation (Landegger et al., 2017). Many studies have shown safe introduction of AAV into perilymphatic spaces without disruption of the barrier between endolymph and perilymph (Akil et al., 2015; Ji et al., 2019). Other commonly used methods were cochleostomy and injection in the posterior semicircular canal (PSC).

**Figure 3:**
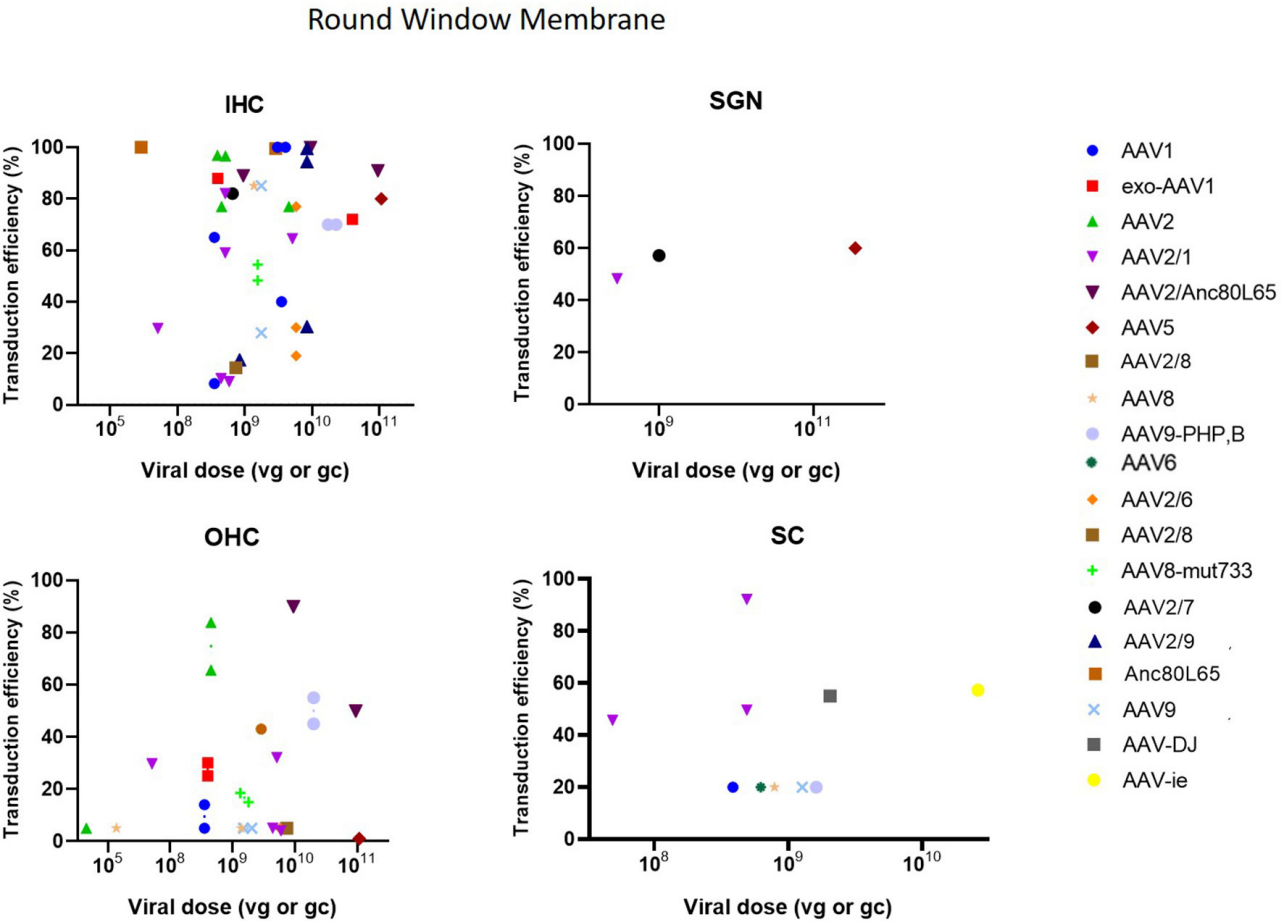
Transduction efficiency of IHC, OHC, SGN and SC according to viral vector dose using the round membrane window approach. Only data from studies that performed inner ear application of AAV in mice were used to plot these graphs. IHC, inner hair cell; OHC, outer hair cell; SGN, spiral ganglion neuron; SC, supporting cell in organ of Corti; AAV, adeno-associated vector; gc, genome copies; vg, viral genomes.

As can be seen in Figure 3, the RWM approach demonstrated a high transduction efficiency in various cell types (HC, SC and SGN), however, an apical to basal gradient can often be observed in gene expression in HC (Yu et al., 2014). Several studies altered the RWM approach to enhance transduction. Three studies combined RWM inoculation with canal fenestration (RWM + CF) (Yoshimura et al., 2018, 2019; Omichi et al., 2020) which resulted in a widespread transduction of IHCs which was remarkably higher as compared to the RWM method in these studies (75 ± 33% compared to around 30%) (Yoshimura et al., 2018, 2019). CF consists of a small fenestration in the posterior semicircular canal to allow a better spread of the injected vector by creating an exit path for perilymph and thereby increasing transduction efficiency (Yoshimura et al., 2018, 2019). Partial digestion of the RWM using collagenase to increase permeability of the RWM was applied in two studies (Wang et al., 2012; Xia et al., 2012). However, transduction efficiency was moderately lower as compared to normal RWM or cochleostomy in these studies (53 ± 33% vs. 68 ± 16%) (Wang et al., 2012; Xia et al., 2012). Notably from these comparisons is also the high variability of the RWM method over different studies.

**Figure 4:**
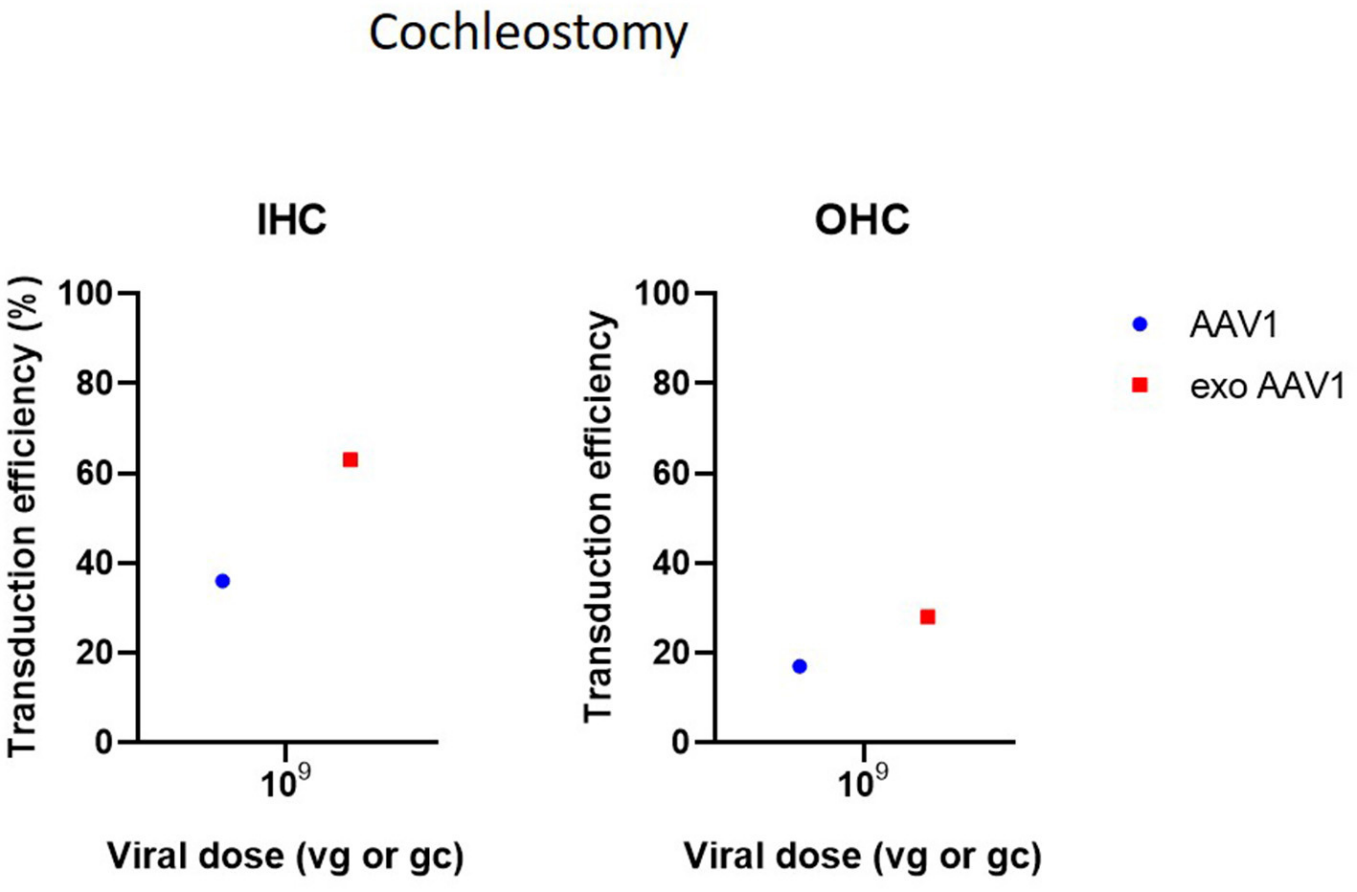
Transduction efficiency of IHC and OHC according to viral vector dose using cochleostomy. Only data from studies that performed inner ear application of AAV in mice were used to plot these graphs. IHC, inner hair cell; OHC, outer hair cell; AAV, adeno-associated vector; gc, genome copies; vg, viral genomes.

Cochleostomy is the second most used delivery method in the included studies. Cochleostomy can be used for both delivery in endolymph or perilymph and shows transduction patterns similar to the RWM. However, several studies have indicated that the risk of surgical trauma is slightly increased in cochleostomy compared to RWM (Chien et al., 2015; Jiam and Limb, 2016). Cochleostomy resulted in successful transgene expression in HCs, SCs and SGNs. As most included studies used Ad vectors for cochleostomies, comparison of transduction efficiency with other studies using AAVs is difficult (Figure 4) (Konishi et al., 2008; Wise et al., 2011; Atkinson et al., 2012; Wang et al., 2012; Gyorgy et al., 2017; Chen et al., 2018).

**Figure 5:**
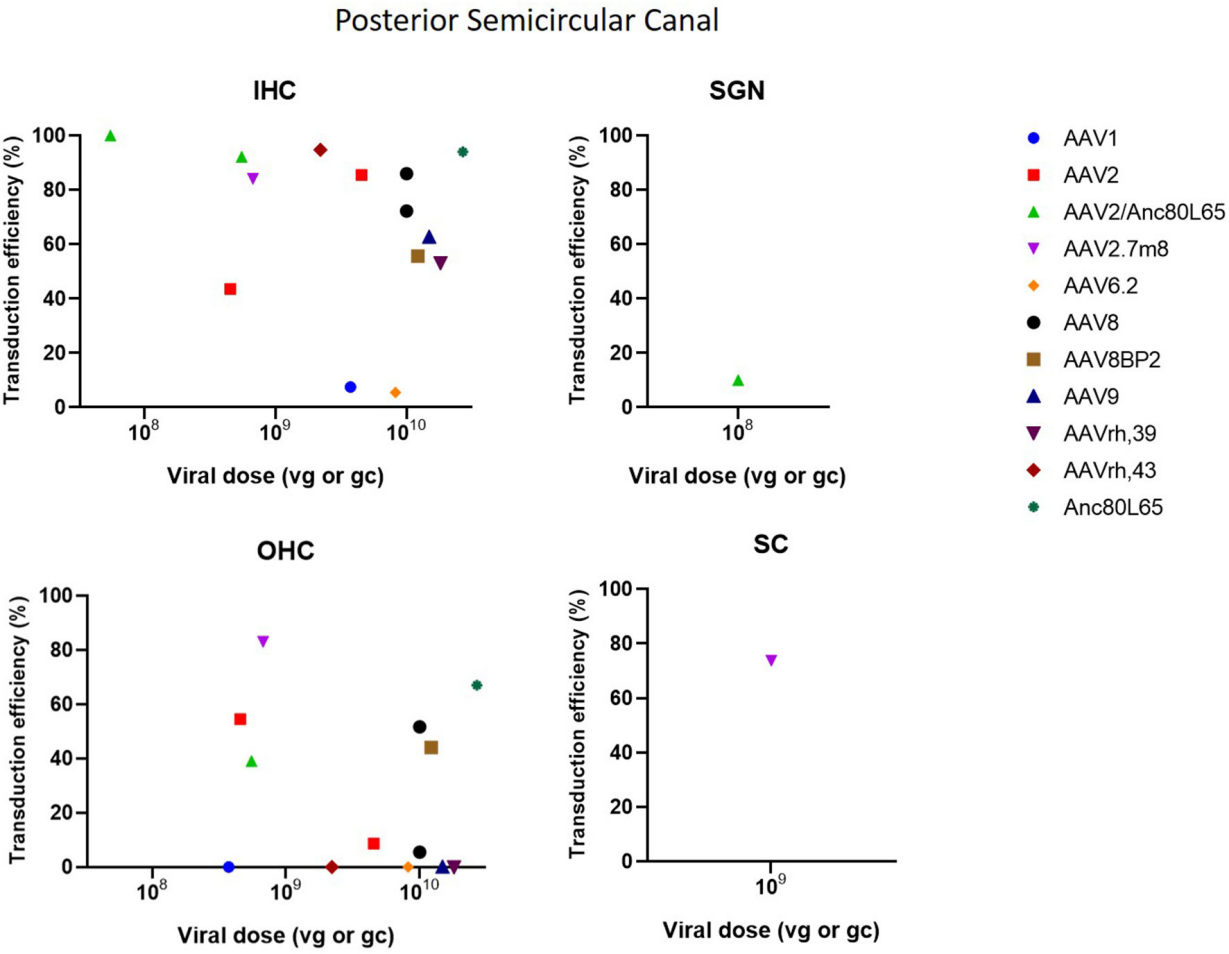
Transduction efficiency of IHC, OHC, SGN and SC according to viral vector dose using the posterior semicircular canal injection. Only data from studies that performed inner ear application of AAV in mice were used to plot these graphs. IHC, inner hair cell; OHC, outer hair cell; SGN, spiral ganglion neuron; SC, supporting cell in organ of Corti; AAV, adeno-associated vector; gc, genome copies; vg, viral genomes.

The posterior semicircular approach (PSC) is used to access the vestibular organs, but is also capable of accessing the cochlea. A significant limitation of the PSC approach is the difficulty to determine whether the vector is injected into the endolymph or perilymph (Talaei et al., 2019). However, because of its relatively easy accessibility, the risk of surgical trauma and thus inner ear damage is decreased, which makes PSC an interesting delivery method (Isgrig and Chien, 2018; Ji et al., 2019). PSC showed to be very efficient at transducing IHC as it resulted in a transduction efficiency of 100% in several studies. Transduction in OHC was moderate with some studies reporting high transduction, whereas other studies demonstrated very low or even no transduction. Furthermore, PSC was also capable in transducing SCs and SGNs, however transduction efficiency was very low (Suzuki et al., 2017; Gyorgy et al., 2018; Tao et al., 2018; Al-Moyed et al., 2019; Ji et al., 2019). These data are represented in Figure 5.

**Figure 6:**
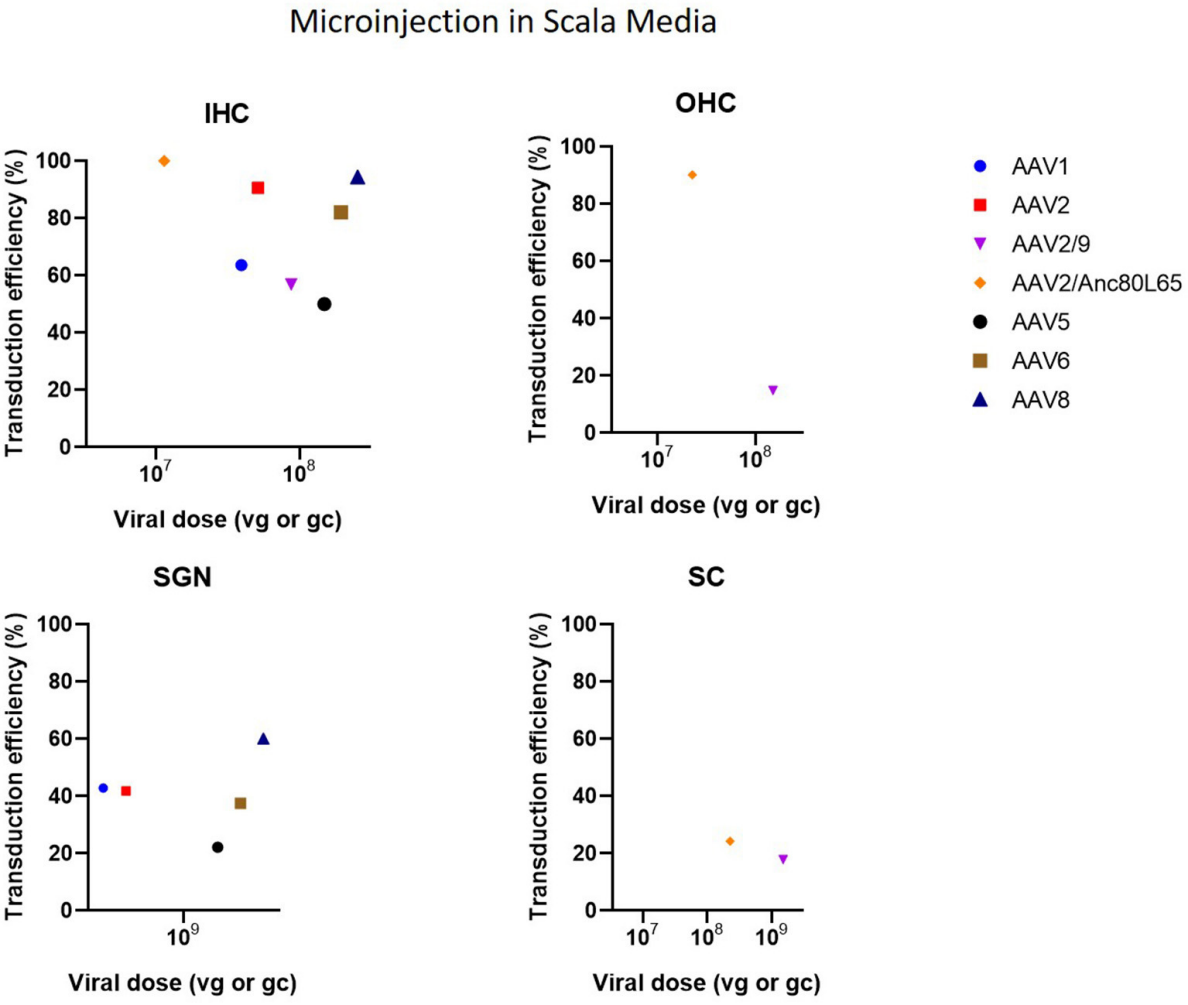
Transduction efficiency of IHC, OHC, SGN and SC according to viral vector dose using microinjection in the scala media. Only data from studies that performed inner ear application of AAV in mice were used to plot these graphs. IHC, inner hair cell; OHC, outer hair cell; SGN, spiral ganglion neuron; SC, supporting cell in organ of Corti; AAV, adeno-associated vector; gc, genome copies; vg, viral genomes.

**Figure 7:**
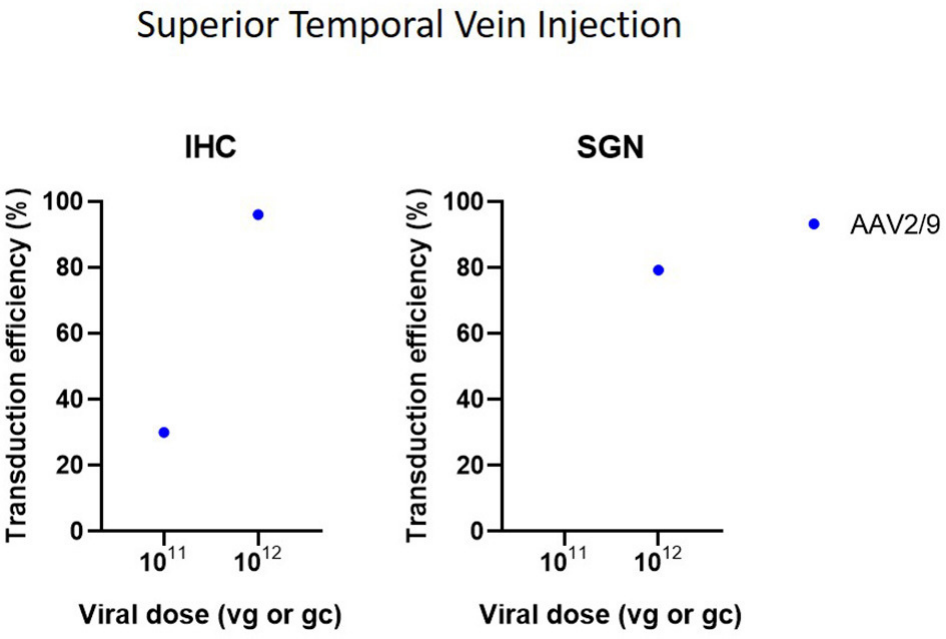
Transduction efficiency of IHC and SGN according to viral vector dose using superior temporal vein injection. Only data from studies that performed inner ear application of AAV in mice were used to plot these graphs. IHC, inner hair cell; SGN, spiral ganglion neuron; AAV, adeno-associated vector; gc, genome copies; vg, viral genomes.

Cochleostomy with injection into the scala media in CBA/CaJ mice demonstrated high transduction efficiency in IHC (50–94%) and mild-to-high transduction efficiency in SGN (22–60%), depending on the AAV serotype (Kilpatrick et al., 2011) (Figure 6). Another study with microinjection into the scala media showed a more abundant gene expression compared to injection to the scala tympani (Gu et al., 2019). However, injection into scala media is surgically more challenging and can cause high-frequency hearing loss (Yoshimura et al., 2018). Injection into the superior temporal vein resulted in high transduction in both HC and SGN in neonatal mice (Shibata et al., 2017) (Figure 7). Transuterine microinjection with adenoviral vector resulted in a widespread transduction in several cell types in the cochlea, however, this expression was weak (Sheffield et al., 2011). However, as these two latter methods were only reported in a single study, and validation of these approaches needs to be confirmed by other studies. Other studies applied inner gene therapy *in utero* with AAV which resulted in high transduction efficiency of IHC, OHC and SGN (Kim et al., 2016; Hu et al., 2020).

### Reporter Gene and Vector Type

Three main types of vectors were used in the included studies: AAV (*n* = 31), Ad (*n* = 7) and lentiviral vectors (LV) (*n* = 2). For AAV vectors, the most commonly used serotype was AAV1 (*n* = 8), followed by AAV2 (*n* = 7) and AAV8 (*n* = 6) and all recombinant vectors used contained AAV2 for the structural replication proteins most often combined with AAV9 (*n* = 5) or Anc80L65 (*n* = 5). Green fluorescent protein (GFP) was used in nearly all studies (*n* = 33) to determine transduction efficiency. AAV2/Anc80L65, AAV2.7m8, AAV2 and AAV8 are the viral vectors to transduce both IHC (43–100%) and OHC (39–83%) most efficiently when injection is performed using the PSC approach (Figure 5). Also, transduction efficiency of SC, using a AAV2.7m8 vector, is reported in only one study where a transduction efficiency of 77% was obtained in SC (Isgrig et al., 2019). Figure 3 shows that AAV1, exo-AAV1, AAV2, AAV2/Anc80L65 and Anc80L65 were able to efficiently transduce IHC (72–100%) when RMW injection was performed. AAV2/1 and AAV-ie are the best viral vectors to transduce SC while Anc80L65 was able to efficiently transduce OHC (90%). Similar to the PSC approach, SGN appear to be the most difficult cell type to transduce (Suzuki et al., 2017). Microinjection in the Scala Media resulted in high transduction efficiency of IHC for all viral vector used, but OHC, SGN and SC were difficult to transduce using this approach (Kilpatrick et al., 2011; Gu et al., 2019). However, the variability among obtained transduction efficiencies using different vector systems applied is extremely high. Consequently, it will be difficult—if not impossible—to select from existing literature the most appropriate vector system without further study.

Adeno-associated viruses (AAVs) are at current the best studied viral vectors in the context of inner ear gene therapy. AAVs are parvoviruses which are non-pathogenic in humans and depend on adenovirus or herpesvirus co-infection for efficient replication. Their genome consists of 4.8 kb single-stranded DNA flanked by two inverted terminal repeats (ITRs) and packaged in a capsid. The capsid, which is comprised of 60 protein subunits organized into an icosahedral structure, is necessary for protection and delivery of the genome (Hastie and Samulski, 2015; Naso et al., 2017). There have been multiple serotypes reported which differ in tropism and cell-binding mechanism (Lee et al., 2018). The use of AAVs has many advantages such as relatively high transduction efficiency, stable gene expression, low immunogenicity and broad tropism (Landegger et al., 2017; Askew and Chien, 2020).

Conventional AAVs have been identified to predominantly transduce IHC, while transduction efficiency is markedly lower in OHC and supporting cells, which is confirmed by our literature survey (63 ± 24% vs. 15 ± 21%). In this context, some AAVs have also been modified to increase transduction efficiency, such as recombinant AAV (rAAV), exosome-associated AAV (exo-AAV), AAV-PHP.B or AAVs with a mutation in surface-exposed capsid tyrosine residues to prevent proteasome-mediated degradation (Xia et al., 2012; Gyorgy et al., 2017; Ivanchenko et al., 2020). Anc80L65 is an AAV designed *in silico* based on a common ancestral sequence of several conventional serotypes, including AAV1, AAV2, AAV8, and AAV9 (Zinn et al., 2015; Hudry et al., 2018). rAAVs combine structural replication proteins of one serotype with capsid proteins of another serotype and can thereby alter vector tropism and efficiency. As rAAVs lack viral DNA, they cannot integrate in the genome and thus after replication loss of transgene expression may occur (Naso et al., 2017). However, rAAV genomes are known to form episomes that are stably expressed in non-dividing cells, which is the case in the inner ear. Nevertheless, even with these new and improved capsid types arising, the general reproducibility of high transduction efficiency still needs to be confirmed by more studies.

Longevity of transgene expression is also important for restoring of hearing function, an issue that may arise when using non-integrating vector delivery systems such rAAV. Longevity of transgene expression also depends on the type of cells targeted. Hair cells and spiral ganglion neurons, two major targets, are post-mitotic and show no regeneration. Therefore, transduction is permanent with no need of re-administration (Askew and Chien, 2020; Maguire and Corey, 2020). Fibrocytes, on the other hand, possess endogenous regenerative capabilities and may thus only require a shorter transduction period (Mizutari, 2014). From the gene therapeutic viewpoint, the main disadvantage of AAVs is the limited packaging capacity of ~4.8 kb. Dual delivery of two transgenes into a single cell may be required to overcome this limitation (Akil et al., 2019b; Al-Moyed et al., 2019; Omichi et al., 2020). As an alternative, LVs and Ads have been used in inner ear gene therapy (Luebke et al., 2009). However, they have been proven not to be effective in preventing sensorineural hearing loss as they are not able to provide efficient transduction of hair cells and demonstrate low expression levels (Gu et al., 2019). Therefore, despite some limitations, currently AAVs are the delivery method of choice.

### Vector Dose

The dose identifies the amount of vector administered. The most common unit of measurement for the viral preparation is genome copies per milliliter (gc/ml) (*n* = 17) or viral genomes per milliliter (vg/ml) (*n* = 10) which are equivalent to each other. Other units of measurement used were: plaque-forming units (pfu) per ml (*n* = 3), focal forming units (ffu) per ml (*n* = 1), Dnase resistant particles (DRP) per ml (*n* = 1), infectious units (IFU) per ml (*n* = 1), total particles per ml (*n* = 1) and optical particle units (OPU) per ml (*n* = 2). Two studies did not provide a unit of measurement and one study made no mention of the dose applied (Yang et al., 2012; Chen et al., 2018). As shown in Figures 3–7, the highest viral vector dose used at each injection route leads to the best transduction efficiency in IHC (10^10^ gc or vg for PSC, 10^11^ gc or vg for RWM, 10^9^ gc or vg for MS and 10^12^ gc or vg for STVI). Transduction of OHC, SGN and SC is not dependent on the viral vector dose as transduction efficiency of these cell types is highly variable among the different injection routes at all viral vector concentrations.

### Promoter

**Figure 8:**
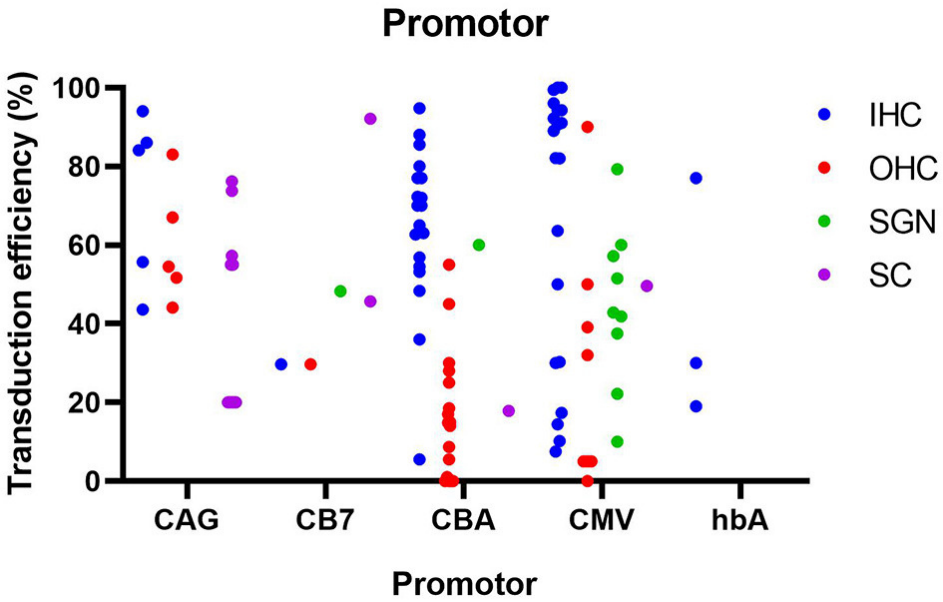
Transduction efficiency of IHC, OHC, SGN and SC according to promoter type in mice. Only data from studies that performed inner ear application of AAV in mice were used to plot this graph. IHC, inner hair cell; OHC, outer hair cell; SGN, spiral ganglion neuron; SC, supporting cell in organ of Corti; CMV, cytomegalovirus; CAG, hybrid promoter; CB7, hybrid promoter; CBA, chicken β-actin; hbA, human β-actin.

For the studies included in this literature review, the CMV promoter was the most commonly used promoter (*n* = 17), followed by the CBA promoter (*n* = 12) and the CAG promoter (*n* = 4) which is composed of the CMV enhancer and the CBA promoter. Other promoters include CB7 (similar to CAG), hBA, ubiquitin and CMV-β-globin, although each was only used in a single study. Furthermore, some of the included studies made no explicit mention of the used promoter (*n* = 3). As shown in Figure 8, the use of the CMV and CBA promoters to drive transgene expression result in the highest transduction efficiency in IHC (resp. 66 ± 33% and 67 ± 22%), but are also capable of transducing other cell types albeit at lower efficiency. Nevertheless, as depicted from the averages given above, variability remains extremely high over different studies, thereby warranting the need for standardized transduction efficiency studies.

CMV and CBA promoters are ubiquitous promoters that can be applied to drive transgene expression in a wide-range of cell types and are therefore most commonly used in gene therapy studies (Gu et al., 2019). The CAG promoter showed the highest transduction efficiency in SC, while also capable of efficiently transducing hair cells, and thus may represent a promoter system to obtain wide spread transgene expression in the inner ear. These promoters and there specifications are represented in Table 4. Other promoters (CB7, hBA, CMV-β-globin) showed intermediate levels of transgene expression, but because of the smaller amount of studies, these results need to be further validated. To further enhance the level of transgene expression, the woodchuck hepatitis virus post-transcriptional (WPRE) regulatory element is often incorporated in the vector cassette (Nist-Lund et al., 2019). WPRE increases the level of transgenic mRNA, resulting in higher expression of the transgene product. It must however be noted that from existing literature, it is highly complicated to correlate transduction efficiency with the actual level of transgene expression, as both are influenced by various parameters, including cell type, vector capsid and promoter/regulatory elements. Like discussed above, more comparative studies will be needed in future research.

**Table 4:** Different promoters used in viral gene therapy.

CAG promoter	C = cytomegalovirus early enhancer element
	A = promoter (first exon and first intron of chicken-β-actin gene)
	G = splice acceptor of the rabbit-β-globin gene
CMV promoter	Cytomegalovirus promoter
CBA promoter	Chicken-β-actin promoter
CB7 promoter	Similar to the CAG promoter, the CB7 promoter also has chicken-β-actin promoters with cytomegalovirus enhancer elements.
CMV-beta-globin promoter	CMV immediate early enhancer + CMV promoter with intron of the rabbit-β-globin gene in the promoter
hBA promoter	Human-β-actin promoter + cytomegalovirus enhancer

### Immunogenicity and Effect on Hearing

Finally, one of the main issues in gene therapy is the safe delivery of a sufficient amount of vector. To assess the safety of viral gene therapy administered in the inner ear, the effect on hearing thresholds and infiltration of inflammatory cells should be investigated. Table 5 gives an overview of the evaluation of hearing function and immunogenicity in the studies included in this systematic review.

**Table 5:** Evaluation of hearing function and immunogenicity.

**Effect on hearing**	**No influence**			**Negative influence**			**Not assessed**		
	**19**			**9**			**8**		
Inflammation	No	Yes	Not assessed	No	Yes	Not assessed	No	Yes	Not assessed
	3	0	16	1	1	7	3	1	4

In Table 5, it is observed that the effect on hearing thresholds was evaluated consistently in 28 out of 36 studies, with nine studies reporting a significant negative effect on hearing function. Only two studies investigated inner ear inflammation after reporting a negative influence on hearing thresholds where one study concluded an infiltration of inflammatory cells in the inner ear caused the observed hearing loss (Isgrig et al., 2019). Only nine out of 36 studies looked at inner ear inflammation where two studies reported an infiltration of inflammatory cells in the cochlea (Isgrig et al., 2019; Ivanchenko et al., 2020). Due to the existence of tight junctions, it has long be thought that the inner ear was an immune-privileged organ similar to the eyes and brain (Fujioka et al., 2014). However, this hypothesis has been challenged arguing that the cochlea is capable of rapidly recruiting immune cells and therefore inducing an immune response (Peeleman et al., 2020). As the cochlea contains resident macrophages in the spiral ligament, spiral ganglion, basilar membrane, and stria vascularis, the immunogenicity and safety of viral gene therapy in the inner ear needs to be more evaluated comprehensively. AAVs have been demonstrated to be less immunogenic when compared to other viral vectors, however, there is a significant pre-existing immunity against AAV. Many people have already been exposed to AAV earlier in life and have generated specific antibodies. Therefore, in clinical trials using AAV, patients are often excluded when they display AAV-specific antibodies (Kotterman and Schaffer, 2014; Naso et al., 2017). This challenge needs to be resolved in order to use gene therapy as an alternative to existing treatments, and we urge this as a highly important topic to include in current pre-clinical gene therapy studies.

## Conclusion

A systematic review was performed to identify preclinical studies reporting viral vector transduction efficiencies in the cochlea, with the purpose of identifying important knowledge gaps. Here we have described several potential issues related to gene therapy in the cochlea including: the use of different animal species and strains which may have a different effect on therapeutic outcome or toxicity, variations in dosage, study design (including randomization, investigators evaluating outcome blinded for treatment), reporting serious adverse events in animals after administration, choice of control group (no treatment, treatment with vehicle without gene therapy), translatability of disorders in animals to human research, etc. These primary outcome measures and end points (including follow-up) should be clearly established before study start and should reflect the human disease state. By comprehensively reporting all these variables, the initial findings can be corroborated by other labs in future research and—most importantly—will avoid or minimize unnecessary adverse effects by gene therapy on possible translation into clinical trials. Nevertheless, cochlear gene therapy is a promising approach to treat and/or prevent sensorineural hearing loss, despite current lack of essential and detailed safety and immunogenicity studies.

## Data Availability Statement

The original contributions presented in the study are included in the article/supplementary material, further inquiries can be directed to the corresponding author/s.

## Author Contributions

DV and NP: study conception and design, data collection, analysis and interpretation of results, draft manuscript preparation. GC study conception and design, analysis and interpretation of results. VR and PP: study conception and design, analysis and interpretation of results, draft manuscript preparation. All authors contributed to the article and approved the submitted version.

## Conflict of Interest

The authors declare that the research was conducted in the absence of any commercial or financial relationships that could be construed as a potential conflict of interest.

## Publisher's Note

All claims expressed in this article are solely those of the authors and do not necessarily represent those of their affiliated organizations, or those of the publisher, the editors and the reviewers. Any product that may be evaluated in this article, or claim that may be made by its manufacturer, is not guaranteed or endorsed by the publisher.

## References

[B1] Ahmed H. Shubina-Oleinik O. Holt J. R. (2017). Emerging gene therapies for genetic hearing loss. J. Assoc. Res. Otolaryngol. 18, 649–670. 10.1007/s10162-017-0634-8 28815315 PMC5612923

[B2] Akil O. Blits B. Lustig L. R. Leake P. A. (2019a). Virally mediated overexpression of glial-derived neurotrophic factor elicits age- and dose-dependent neuronal toxicity and hearing loss. Hum. Gene Ther. 30, 88–105. 10.1089/hum.2018.028 30183384 PMC6343370

[B3] Akil O. Dyka F. Calvet C. Emptoz A. Lahlou G. Nouaille S.. (2019b). Dual AAV-mediated gene therapy restores hearing in a DFNB9 mouse model. Proc. Natl. Acad. Sci. U. S. A. 116, 4496–4501. 10.1073/pnas.1817537116 30782832 PMC6410774

[B4] Akil O. Rouse S. L. Chan D. K. Lustig L. R. (2015). Surgical method for virally mediated gene delivery to the mouse inner ear through the round window membrane. J. Vis. Exp. 97, 52187. 10.3791/52187 25867531 PMC4401361

[B5] Akil O. Seal R. P. Burke K. Wang C. Alemi A. During M.. (2012). Restoration of hearing in the VGLUT3 knockout mouse using virally mediated gene therapy. Neuron 75, 283–293. 10.1016/j.neuron.2012.05.019 22841313 PMC3408581

[B6] Al-Moyed H. Cepeda A. P. Jung S. Moser T. Kugler S. Reisinger E. (2019). A dual-AAV approach restores fast exocytosis and partially rescues auditory function in deaf otoferlin knock-out mice. EMBO Mol. Med. 11:e9396. 10.15252/emmm.201809396 30509897 PMC6328916

[B7] Askew C. Chien W. W. (2020). Adeno-associated virus gene replacement for recessive inner ear dysfunction: progress and challenges. Hear. Res. 394:107947. 10.1016/j.heares.2020.107947 32247629 PMC7939749

[B8] Askew C. Rochat C. Pan B. Asai Y. Ahmed H. Child E.. (2015). Tmc gene therapy restores auditory function in deaf mice. Sci. Transl. Med. 7:295ra108. 10.1126/scitranslmed.aab1996 26157030 PMC7298700

[B9] Atkinson P. J. Wise A. K. Flynn B. O. Nayagam B. A. Hume C. R. O'Leary S. J.. (2012). Neurotrophin gene therapy for sustained neural preservation after deafness. PLoS ONE 7:e52338. 10.1371/journal.pone.0052338 23284995 PMC3524079

[B10] Bond M. Elston J. Mealing S. Anderson R. Weiner G. Taylor R.. (2010). Systematic reviews of the effectiveness and cost-effectiveness of multi-channel unilateral cochlear implants for adults. Clin. Otolaryngol. 35, 87–96. 10.1111/j.1749-4486.2010.02098.x 20500577

[B11] Bond M. Mealing S. Anderson R. Elston J. Weiner G. Taylor R. S.. (2009). The effectiveness and cost-effectiveness of cochlear implants for severe to profound deafness in children and adults: a systematic review and economic model. Health Technol. Assess. 13, 1–330. 10.3310/hta13440 19799825

[B12] Bowl M. R. Dawson S. J. (2015). The mouse as a model for age-related hearing loss - a mini-review. Gerontology 61, 149–157. 10.1159/000368399 25471225

[B13] Burton J. A. Valero M. D. Hackett T. A. Ramachandran R. (2019). The use of nonhuman primates in studies of noise injury and treatment. J. Acoust. Soc. Am. 146:3770. 10.1121/1.5132709 31795680 PMC6881191

[B14] Chen H. Xing Y. Xia L. Chen Z. Yin S. Wang J. (2018). AAV-mediated NT-3 overexpression protects cochleae against noise-induced synaptopathy. Gene Ther. 25, 251–259. 10.1038/s41434-018-0012-0 29535374 PMC6062503

[B15] Chien W. W. Isgrig K. Roy S. Belyantseva I. A. Drummond M. C. May L. A.. (2016). Gene therapy restores hair cell stereocilia morphology in inner ears of deaf whirler mice. Mol. Ther. 24, 17–25. 10.1038/mt.2015.150 26307667 PMC4754541

[B16] Chien W. W. McDougald D. S. Roy S. Fitzgerald T. S. Cunningham L. L. (2015). Cochlear gene transfer mediated by adeno-associated virus: comparison of two surgical approaches. Laryngoscope 125, 2557–2564. 10.1002/lary.25317 25891801

[B17] Davis A. C. Hoffman H. J. (2019). Hearing Loss: Rising Prevalence and Impact. Available online at: https://www.who.int/bulletin/volumes/97/10/19-224683/en/ (accessed May 15, 2021). 10.2471/BLT.19.224683 PMC6796666 31656325

[B18] Emptoz A. Michel V. Lelli A. Akil O. Boutet de Monvel J. Lahlou G.. (2017). Local gene therapy durably restores vestibular function in a mouse model of Usher syndrome type 1G. Proc. Natl. Acad. Sci. U. S. A. 114, 9695–9700. 10.1073/pnas.1708894114 28835534 PMC5594693

[B19] Fujioka M. Okano H. Ogawa K. (2014). Inflammatory and immune responses in the cochlea: potential therapeutic targets for sensorineural hearing loss. Front. Pharmacol. 5:287. 10.3389/fphar.2014.00287 25566079 PMC4274906

[B20] Gao X. Tao Y. Lamas V. Huang M. Yeh W. H. Pan B.. (2018). Treatment of autosomal dominant hearing loss by in vivo delivery of genome editing agents. Nature 553, 217–221. 10.1038/nature25164 29258297 PMC5784267

[B21] Gu X Chai R. Guo L. Dong B. Li W. Shu Y.. (2019). Transduction of adeno-associated virus vectors targeting hair cells and supporting cells in the neonatal mouse cochlea. Front. Cell Neurosci. 13:8. 10.3389/fncel.2019.00008 30733670 PMC6353798

[B22] Gyorgy B. Meijer E. J. Ivanchenko M. V. Tenneson K. Emond F. Hanlon K. S.. (2018). Gene transfer with AAV9-PHP.B rescues hearing in a mouse model of usher syndrome 3A and transduces hair cells in a non-human primate. Mol. Ther. Methods Clin. Dev. 13, 1–13. 10.1016/j.omtm.2018.11.003 30581889 PMC6297893

[B23] Gyorgy B. Nist-Lund C. Pan B. Asai Y. Karavitaki K. D. Kleinstiver B. P.. (2019). Allele-specific gene editing prevents deafness in a model of dominant progressive hearing loss. Nat. Med. 25, 1123–1130. 10.1038/s41591-019-0500-9 31270503 PMC6802276

[B24] Gyorgy B. Sage C. Indzhykulian A. A. Scheffer D. I. Brisson A. R. Tan S.. (2017). Rescue of hearing by gene delivery to inner-ear hair cells using exosome-associated AAV. Mol. Ther. 25, 379–391. 10.1016/j.ymthe.2016.12.010 28082074 PMC5368844

[B25] Hastie E. Samulski R. J. (2015). Adeno-associated virus at 50: a golden anniversary of discovery, research, and gene therapy success–a personal perspective. Hum. Gene Ther. 26, 257–265. 10.1089/hum.2015.025 25807962 PMC4442590

[B26] Hooijmans C. R. Rovers M. M. de Vries R. B. Leenaars M. Ritskes-Hoitinga M. Langendam M. W. (2014). SYRCLE's risk of bias tool for animal studies. BMC Med. Res. Methodol. 14:43. 10.1186/1471-2288-14-43 24667063 PMC4230647

[B27] Hoppe U. Hesse G. (2017). Hearing aids: indications, technology, adaptation, and quality control. GMS Curr. Top. Otorhinolaryngol. Head Neck Surg. 16:Doc08. 10.3205/cto000147 29279726 PMC5738937

[B28] Hu C. J. Lu Y. C. Tsai Y. H. Cheng H. Y. Takeda H. Huang C. Y.. (2020). Efficient in utero gene transfer to the mammalian inner ears by the synthetic adeno-associated viral vector Anc80L65. Mol. Ther. Methods Clin. Dev. 18, 493–500. 10.1016/j.omtm.2020.06.019 32775487 PMC7390729

[B29] Hudry E. Andres-Mateos E. Lerner E. P. Volak A. Cohen O. Hyman B. T.. (2018). Efficient gene transfer to the central nervous system by single-stranded Anc80L65. Mol. Ther. Methods Clin. Dev. 10, 197–209. 10.1016/j.omtm.2018.07.006 30109242 PMC6083902

[B30] Iizuka T. Kamiya K. Gotoh S. Sugitani Y. Suzuki M. Noda T.. (2015). Perinatal Gjb2 gene transfer rescues hearing in a mouse model of hereditary deafness. Hum. Mol. Genet. 24, 3651–3661. 10.1093/hmg/ddv109 25801282

[B31] Isgrig K. Chien W. W. (2018). Posterior semicircular canal approach for inner ear gene delivery in neonatal mouse. J. Vis. Exp. 133, 56648. 10.3791/56648 29553564 PMC5931426

[B32] Isgrig K. McDougald D. S. Zhu J. Wang H. J. Bennett J. Chien W. W. (2019). AAV2.7m8 is a powerful viral vector for inner ear gene therapy. Nat. Commun. 10:427. 10.1038/s41467-018-08243-1 30683875 PMC6347594

[B33] Ivanchenko M. V. Hanlon K. S. Devine M. K. Tenneson K. Emond F. Lafond J. F.. (2020). Preclinical testing of AAV9-PHP.B for transgene expression in the non-human primate cochlea. Hear. Res. 394:107930. 10.1016/j.heares.2020.107930 32145977 PMC7415510

[B34] Ji X. J. Chen W. Wang X. Zhang Y. Liu Q. Guo W. W.. (2019). Canalostomy is an ideal surgery route for inner ear gene delivery in big animal model. Acta Otolaryngol. 139, 939–947. 10.1080/00016489.2019.1654130 31486693

[B35] Jiam N. T. Limb C. J. (2016). The impact of round window vs cochleostomy surgical approaches on interscalar excursions in the cochlea: preliminary results from a flat-panel computed tomography study. World J. Otorhinolaryngol. Head Neck Surg. 2, 142–147. 10.1016/j.wjorl.2016.07.001 29204559 PMC5698540

[B36] Kilpatrick L. A. Li Q. Yang J. Goddard J. C. Fekete D. M. Lang H. (2011). Adeno-associated virus-mediated gene delivery into the scala media of the normal and deafened adult mouse ear. Gene Ther. 18, 569–578. 10.1038/gt.2010.175 21209625 PMC3085601

[B37] Kim M. A. Cho H. J. Bae S. H. Lee B. Oh S. K. Kwon T. J.. (2016). Methionine sulfoxide reductase B3-targeted in utero gene therapy rescues hearing function in a mouse model of congenital sensorineural hearing loss. Antioxid. Redox Signal 24, 590–602. 10.1089/ars.2015.6442 26649646 PMC4840920

[B38] Konishi M. Kawamoto K. Izumikawa M. Kuriyama H. Yamashita T. (2008). Gene transfer into guinea pig cochlea using adeno-associated virus vectors. J. Gene Med. 10, 610–618. 10.1002/jgm.1189 18338819

[B39] Kotterman M. A. Schaffer D. V. (2014). Engineering adeno-associated viruses for clinical gene therapy. Nat. Rev. Genet. 15, 445–451. 10.1038/nrg3742 24840552 PMC4393649

[B40] Landegger L. D. Pan B. Askew C. Wassmer S. J. Gluck S. D. Galvin A.. (2017). A synthetic AAV vector enables safe and efficient gene transfer to the mammalian inner ear. Nat. Biotechnol. 35, 280–284. 10.1038/nbt.3781 28165475 PMC5340646

[B41] Landsberger D. M. Vermeire K. Claes A. Van Rompaey V. Van de Heyning P. (2016). Qualities of single electrode stimulation as a function of rate and place of stimulation with a cochlear implant. Ear Hear. 37, e149–e159. 10.1097/AUD.0000000000000250 26583480 PMC4844766

[B42] Leake P. A. Rebscher S. J. Dore C. Akil O. (2019). AAV-mediated neurotrophin gene therapy promotes improved survival of cochlear spiral ganglion neurons in neonatally deafened cats: comparison of AAV2-hBDNF and AAV5-hGDNF. J. Assoc. Res. Otolaryngol. 20, 341–361. 10.1007/s10162-019-00723-5 31222416 PMC6646500

[B43] Lee E. J. Guenther C. M. Suh J. (2018). Adeno-Associated Virus (AAV) vectors: rational design strategies for capsid engineering. Curr. Opin. Biomed. Eng. 7, 58–63. 10.1016/j.cobme.2018.09.004 31106283 PMC6516759

[B44] Leenaars M. Hooijmans C. R. van Veggel N. ter Riet G. Leeflang M. Hooft L.. (2012). A step-by-step guide to systematically identify all relevant animal studies. Lab. Anim. 46, 24–31. 10.1258/la.2011.011087 22037056 PMC3265183

[B45] Lei L. Han D. (2010). Efficient transduction of spiral ganglion cells using adenovirus type 5 vector in the rat. Acta Otolaryngol. 130, 810–814. 10.3109/00016480903510742 20205620

[B46] Luebke A. E. Rova C. Von Doersten P. G. Poulsen D. J. (2009). Adenoviral and AAV-mediated gene transfer to the inner ear: role of serotype, promoter, and viral load on in vivo and in vitro infection efficiencies. Adv. Otorhinolaryngol. 66, 87–98. 10.1159/000218209 19494574

[B47] MaguireC. A.CoreyD. P. (2020). Viral vectors for gene delivery to the inner ear. Hear. Res. 394:107927. 10.1016/j.heares.2020.10792732199720

[B48] Mignini L. E. Khan K. S. (2006). Methodological quality of systematic reviews of animal studies: a survey of reviews of basic research. BMC Med. Res. Methodol. 6:10. 10.1186/1471-2288-6-10 16533396 PMC1435907

[B49] Mizutari K. (2014). Spontaneous recovery of cochlear fibrocytes after severe degeneration caused by acute energy failure. Front. Pharmacol. 5:198. 10.3389/fphar.2014.00198 25206337 PMC4143613

[B50] Muller M. K. Jovanovic S. Keine C. Radulovic T. Rubsamen R. Milenkovic I. (2019). Functional development of principal neurons in the anteroventral cochlear nucleus extends beyond hearing onset. Front. Cell Neurosci. 13:119. 10.3389/fncel.2019.00119 30983974 PMC6447607

[B51] Naert G. Pasdelou M. P. Le Prell C. G. (2019). Use of the guinea pig in studies on the development and prevention of acquired sensorineural hearing loss, with an emphasis on noise. J. Acoust. Soc. Am. 146:3743. 10.1121/1.5132711 31795705 PMC7195866

[B52] Naso M. F. Tomkowicz B. Perry W. L. 3rd Strohl W. R. (2017). Adeno-Associated Virus (AAV) as a vector for gene therapy. BioDrugs 31, 317–334. 10.1007/s40259-017-0234-5 28669112 PMC5548848

[B53] Nist-Lund C. A. Pan B. Patterson A. Asai Y. Chen T. Zhou W.. (2019). Improved TMC1 gene therapy restores hearing and balance in mice with genetic inner ear disorders. Nat. Commun. 10:236. 10.1038/s41467-018-08264-w 30670701 PMC6342993

[B54] Ohlemiller K. K. (2019). Mouse methods and models for studies in hearing. J. Acoust. Soc. Am. 146:3668. 10.1121/1.5132550 31795658

[B55] Ohlemiller K. K. Jones S. M. Johnson K. R. (2016). Application of mouse models to research in hearing and balance. J. Assoc. Res. Otolaryngol. 17, 493–523. 10.1007/s10162-016-0589-1 27752925 PMC5112220

[B56] Omichi R. Shibata S. B. Morton C. C. Smith R. J. H. (2019). Gene therapy for hearing loss. Hum. Mol. Genet. 28, R65–R79 10.1093/hmg/ddz129 31227837 PMC6796998

[B57] Omichi R. Yoshimura H. Shibata S. B. Vandenberghe L. H. Smith R. J. H. (2020). Hair cell transduction efficiency of single- and dual-AAV serotypes in adult murine cochleae. Mol. Ther. Methods Clin. Dev. 17, 1167–1177. 10.1016/j.omtm.2020.05.007 32518805 PMC7270144

[B58] Pan B. Askew C. Galvin A. Heman-Ackah S. Asai Y. Indzhykulian A. A.. (2017). Gene therapy restores auditory and vestibular function in a mouse model of Usher syndrome type 1c. Nat. Biotechnol. 35, 264–272. 10.1038/nbt.3801 28165476 PMC5340578

[B59] Peeleman N. Verdoodt D. Ponsaerts P. Van Rompaey V. (2020). On the role of fibrocytes and the extracellular matrix in the physiology and pathophysiology of the spiral ligament. Front. Neurol. 11:580639. 10.3389/fneur.2020.580639 33193034 PMC7653186

[B60] Peters J. L. Sutton A. J. Jones D. R. Rushton L. Abrams K. R. (2006). A systematic review of systematic reviews and meta-analyses of animal experiments with guidelines for reporting. J. Environ. Sci. Health B. 41, 1245–1258. 10.1080/03601230600857130 16923604

[B61] Praetorius M. Brough D. E. Hsu C. Plinkert P. K. Pfannenstiel S. C. Staecker H. (2009). Adenoviral vectors for improved gene delivery to the inner ear. Hear. Res. 248, 31–38. 10.1016/j.heares.2008.11.009 19105978 PMC2679534

[B62] Reis A. D. Dalmolin S. P. Dallegrave E. (2017). Modelos animais para avaliação auditiva: revisão de literatura. Revista CEFAC 19, 417–428. 10.1590/1982-021620171932117

[B63] Ren Y. Landegger L. D. Stankovic K. M. (2019). Gene therapy for human sensorineural hearing loss. Front. Cell Neurosci. 13:323. 10.3389/fncel.2019.00323 31379508 PMC6660246

[B64] Sheffield A. M. Gubbels S. P. Hildebrand M. S. Newton S. S. Chiorini J. A. Di Pasquale G.. (2011). Viral vector tropism for supporting cells in the developing murine cochlea. Hear. Res. 277, 28–36. 10.1016/j.heares.2011.03.016 21530627 PMC3137760

[B65] Shi X. Wu N. Zhang Y. Guo W. Lin C. Yang S. (2017). Adeno-associated virus transformation into the normal miniature pig and the normal guinea pigs cochlea via scala tympani. Acta Otolaryngol. 137, 910–916. 10.1080/00016489.2017.1312015 28471702

[B66] Shibata S. B. Yoshimura H. Ranum P. T. Goodwin A. T. Smith R. J. H. (2017). Intravenous rAAV2/9 injection for murine cochlear gene delivery. Sci. Rep. 7:9609. 10.1038/s41598-017-09805-x 28852025 PMC5575199

[B67] Suen J. J. Marrone N. Han H. R. Lin F. R. Nieman C. L. (2019). Translating public health practices: community-based approaches for addressing hearing health care disparities. Semin. Hear. 40, 37–48. 10.1055/s-0038-1676782 30728648 PMC6363549

[B68] Suzuki J. Hashimoto K. Xiao R. Vandenberghe L. H. Liberman M. C. (2017). Cochlear gene therapy with ancestral AAV in adult mice: complete transduction of inner hair cells without cochlear dysfunction. Sci. Rep. 7:45524. 10.1038/srep45524 28367981 PMC5377419

[B69] Taiber S. Cohen R. Yizhar-Barnea O. Sprinzak D. Holt J. R. Avraham K. B. (2021). Neonatal AAV gene therapy rescues hearing in a mouse model of SYNE4 deafness. EMBO Mol. Med. 13:e13259. 10.15252/emmm.202013259 33350593 PMC7863404

[B70] Talaei S. Schnee M. E. Aaron K. A. Ricci A. J. (2019). dye tracking following posterior semicircular canal or round window membrane injections suggests a role for the cochlea aqueduct in modulating distribution. Front. Cell Neurosci. 13:471. 10.3389/fncel.2019.00471 31736710 PMC6833940

[B71] Tan F. Chu C. Qi J. Li W. You D. Li K.. (2019). AAV-ie enables safe and efficient gene transfer to inner ear cells. Nat. Commun. 10:3733. 10.1038/s41467-019-11687-8 31427575 PMC6700137

[B72] Tao Y. Huang M. Shu Y. Ruprecht A. Wang H. Tang Y.. (2018). Delivery of adeno-associated virus vectors in adult mammalian inner-ear cell subtypes without auditory dysfunction. Hum. Gene Ther. 29, 492–506. 10.1089/hum.2017.120 29130354 PMC5909114

[B73] Vickers D. De Raeve L. Graham J. (2016). International survey of cochlear implant candidacy. Cochlear Implants Int. 17, 36–41. 10.1080/14670100.2016.1155809 27099109

[B74] Wang H. Murphy R. Taaffe D. Yin S. Xia L. Hauswirth W. W.. (2012). Efficient cochlear gene transfection in guinea-pigs with adeno-associated viral vectors by partial digestion of round window membrane. Gene Ther. 19, 255–263. 10.1038/gt.2011.91 21697953 PMC3641535

[B75] Wang Y. Sun Y. Chang Q. Ahmad S. Zhou B. Kim Y.. (2013). Early postnatal virus inoculation into the scala media achieved extensive expression of exogenous green fluorescent protein in the inner ear and preserved auditory brainstem response thresholds. J. Gene Med. 15, 123–133. 10.1002/jgm.2701 23413036

[B76] WHO (2013). Priority Medicines for Europe and the World Update Report. Available online at: http://www.who.int/medicines/areas/priority_medicines/Ch6_21Hearing.pdf (accessed May 15, 2021).

[B77] Wise A. K. Tu T. Atkinson P. J. Flynn B. O. Sgro B. E. Hume C.. (2011). The effect of deafness duration on neurotrophin gene therapy for spiral ganglion neuron protection. Hear. Res. 278, 69–76. 10.1016/j.heares.2011.04.010 21557994 PMC3152700

[B78] Xia L. Yin S. Wang J. (2012). Inner ear gene transfection in neonatal mice using adeno-associated viral vector: a comparison of two approaches. PLoS ONE 7:e43218. 10.1371/journal.pone.0043218 22912830 PMC3422324

[B79] Yang S. M. Chen W. Guo W. W. Jia S. Sun J. H. Liu H. Z.. (2012). Regeneration of stereocilia of hair cells by forced Atoh1 expression in the adult mammalian cochlea. PLoS ONE 7:e46355. 10.1371/journal.pone.0046355 23029493 PMC3459923

[B80] Yoshimura H. Shibata S. B. Ranum P. T. Moteki H. Smith R. J. H. (2019). Targeted allele suppression prevents progressive hearing loss in the mature murine model of human TMC1 deafness. Mol Ther. 27, 681–690. 10.1016/j.ymthe.2018.12.014 30686588 PMC6403483

[B81] Yoshimura H. Shibata S. B. Ranum P. T. Smith R. J. H. (2018). Enhanced viral-mediated cochlear gene delivery in adult mice by combining canal fenestration with round window membrane inoculation. Sci. Rep. 8:2980. 10.1038/s41598-018-21233-z 29445157 PMC5812997

[B82] Yu Q. Wang Y. Chang Q. Wang J. Gong S. Li H.. (2014). Virally expressed connexin26 restores gap junction function in the cochlea of conditional Gjb2 knockout mice. Gene Ther. 21, 71–80. 10.1038/gt.2013.59 24225640 PMC3881370

[B83] Zinn E. Pacouret S. Khaychuk V. Turunen H. T. Carvalho L. S. Andres-Mateos E.. (2015). *In Silico* reconstruction of the viral evolutionary lineage yields a potent gene therapy vector. Cell Rep. 12, 1056–1068. 10.1016/j.celrep.2015.07.019 26235624 PMC4536165

